# The Associations of SCFA with Anthropometric Parameters and Carbohydrate Metabolism in Pregnant Women

**DOI:** 10.3390/ijms21239212

**Published:** 2020-12-03

**Authors:** Małgorzata Szczuko, Justyna Kikut, Dominika Maciejewska, Danuta Kulpa, Zbigniew Celewicz, Maciej Ziętek

**Affiliations:** 1 Department of Human Nutrition and Metabolomics, Pomeranian Medical University in Szczecin, Broniewskiego 24, 71-460 Szczecin, Poland; justyna.kikut@pum.edu.pl (J.K.); domi.maciejka@wp.pl (D.M.); 2 Department of Genetics, Plant Breeding and Biotechnology, Faculty of Environmental Management and Agriculture, West Pomeranian University of Technology, Słowackiego 17, 71-434 Szczecin, Poland; danuta.kulpa@zut.edu.pl; 3 Department of Perinatology, Obstetrics and Gynecology, Pomeranian Medical University in Szczecin, Siedlecka 2, 72-010 Police, Poland; sekr.perinat@spsk1.szn.pl (Z.C.); maciej.zietek@pum.edu.pl (M.Z.)

**Keywords:** SCFA, pregnancy, propionic acid, butyric acid, caproic acid, heptanoic acid

## Abstract

Short-chain fatty acids (SCFAs) mediate the transmission of signals between the microbiome and the immune system and are responsible for maintaining balance in the anti-inflammatory reaction. Pregnancy stages alter the gut microbiota community structure, which also synthesizes SCFAs. The study involved 90 pregnant women, divided into two groups: 48 overweight/obese pregnant women (OW) and 42 pregnant women with normal BMI (CG). The blood samples for glucose, insulin, and HBA1c were analyzed as well as stool samples for SCFA isolation (C2:0; C3:0; C4:0i; C4:0n; C5:0i; C5:0n; C6:0i; C6:0n) using gas chromatography. The SCFA profile in the analyzed groups differed significantly. A significant positive correlation between C2:0, C3:0, C4:0n and anthropometric measurements, and between C2:0, C3:0, C4:0n, and C5:0n and parameters of carbohydrate metabolism was found. SCFA levels fluctuate during pregnancy and the course of pregnancy and participate in the change in carbohydrate metabolism as well. The influence of C2:0 during pregnancy on anthropometric parameters was visible in both groups (normal weight and obese). Butyrate and propionate regulate glucose metabolism by stimulating the process of intestinal gluconeogenesis. The level of propionic acid decreases with the course of pregnancy, while its increase is characteristic of obese women, which is associated with many metabolic adaptations. Propionic and linear caproic acid levels can be an important critical point in maintaining lower anthropometric parameters during pregnancy.

## 1. Introduction

### 1.1. SCFAs—Synthesis, Occurrence and Role

Interest in dietary fiber, gut fermentation, and probiotics has led to studies on short chain fatty acids (SCFA), such as acetic, propionic, and n-butyric acids produced in the large intestine by gut bacteria. SCFA arouse interest in the context of issues related to the microbiota, which constitute the group of microorganisms including bacteria, archaea, viruses, and fungi living in our body. It can be estimated that over 1000 species of microorganisms reside in our intestines, the vast majority of which are bacteria [1,2]. Not only species diversity, but also the estimated number of around one hundred billion cells, represent a great potential influencing the health and disease status [1]. The gut microbiota can regulate about 10% of the host’s transcriptome, i.e., genes involved in the immune response, proliferation, and metabolism, which is especially important in pregnant women [3]. The mentioned microbiota produce metabolites, including SCFAs, B vitamins, hydrochloride, 3-hydroxy-3-methylbutyric acid (HMB), trimethylamine (TMAO), and others, which significantly affect the balance and disturbance of the homeostasis of a human body [4]. Microbial metabolites or components may be related to diseases such as allergic and immune disorders (SCFAs, B vitamins), colorectal cancer (SCFAs, B vitamins, N1,N12-diacetylspermine), bacterial vaginosis and other sexually transmitted infections (polyamines, HBP), hypertension and inflammation, preterm labour (SCFAs), cardiovascular diseases, obesity and metabolic syndrome (TMAO), and type 2 diabetes (TMAO) [4]. Clinical evidence confirms the influence of SCFAs not only on the functioning of the colon, but also the intestinal immune system and metabolic process efficiency [2,5]. SCFAs are produced by carbohydrate fermentation in the large intestine (colon) that have not been digested in the small intestine [6,7]. Branched SCFAs (BSCFAs), e.g., isobutyric and isovaleric acid, are generated by the fermentation of branched amino acids (valine, leucine, and isoleucine) generated from undigested protein reaching the colon. Acetic acid, propionic acid, and butyric acid can be produced by gut microbes generated from colonic fermentation of dietary fibers [8,9]. As result of non-digestible carbohydrate bacterial fermentation in the colon, butyrate may constitute a major energy source for epithelial cells and influence the intestinal epithelial barrier function with immune function modulation [2]. There is a strong relationship between the intestinal microflora, its metabolites (mainly SCFAs), and ingested food [10]. SCFAs include acetic acid (C2: 0), propionic acid (C3: 0), butyric acid (C4: 0), valeric acid (C5: 0), and caproic acid (C6: 0), of which butyrate, acetate, and propionate are the most common SCFAs in our body as they account for ≥95% of the total SCFA pool [10]. Up to 10% of the daily caloric requirements is provided by SCFAs, which are used as a source of energy [10]. A small amount of C5: 0-C6: 0 acids may come from food such as herbs. The amount of SCFAs in the colon is maintained at the level of 60–150 mmol/kg, and the ratios are 60:25:15 acetate, propionate, and butyrate, respectively [11]. Studies on fecal gut microbiota have shown that at a pH 5.5, butyrate-producing bacteria, such as *Roseburia* spp. and *Faecalibacterium prausnitzii* belonging to the Firmicutes type, account for 20% of the total bacterial count. In the distal part of the large intestine, bacteria producing C3:0 and C2:0 are dominant, while the production of C4:0 decreases [12].

The large intestine is inhabited mainly by anaerobic bacteria, e.g., bacteria of the genera *Bacteroides* spp., *Bifidobacterium* spp., lactobacilli, streptococci, and eubacteria [13]. *Bacteroides* are one of the major lineages of bacteria, whose abundance reaches up to 30% of the entire bacterial population. The increase in SCFA production through bacterial fermentation of microbiota causes a pH decrease [14]. Thus, the overgrowth of pH-sensitive pathogenic bacteria may be prevented by lower pH values and raised SCFA [14]. The gut microbiota can be considered as a “metabolic organ” which, through the SCFA action, may influence many processes and play an important role in metabolic dysfunction during gestation [15]. The disruption of homeostasis contributes to a variety of metabolic disorders to which pregnant women are particularly exposed, due to the exceptional physiological state of gestation. It is estimated that about 60% of SCFAs diffuse into the lamina propria from the intestinal lumen through the intestinal epithelium, and about 5% of SCFAs are excreted in the faeces [10]. SCFAs are a source of energy for intestinal epithelial cells [10,16,17,18]. Colonocytes promote butyrate, which constitute the main energetic substrate [9]. This process takes place even in the presence of other substrates such as glucose or glutamine, meaning that colonocytes prefer butyrate. Butyrate provides about 70% of the energy requirements of the colon cells. It is an essential component for the development and differentiation of colonocytes [19]. Hormonal and immunological changes in pregnancy may strongly influence the formation of intestinal bacterial [20,21].

### 1.2. The Role of SCFA in Pregnancy

Pregnancy is a dynamic period during which physiological, anatomical, and metabolic changes occur [22,23]. Metabolic and immunological shifts may be compared to the metabolic syndrome [24,25].

At the beginning of pregnancy, an increase in insulin release from the pancreas is observed, with initially unchanged tissue sensitivity to its effects. Only in the late first-trimester and midpregnancy does the insulin sensitivity begin to decline and is finally reduced [26]. The last trimester of pregnancy is defined as a diabetogenic state, due to increased glucose levels in plasma of a pregnant woman [23]. The first two trimesters of pregnancy are a period of anabolic transformations with intense lipogenesis. In the third trimester, a catabolic phase is observed with increased lipolysis, and increased serum free fatty acids and VLDL lipoproteins. The diabetogenic effects of hormones such as 17 beta estradiol, progesterone, prolactin, placental lactogen and an increase in adipose tissue lead to pregnancy insulin resistance. However, glucose metabolism in pregnancy may also be regulated by the gut microbiota. It has been demonstrated that the abundance of the Actinobacteria genus *Collinsella* is positively correlated with circulating insulin [27]. The research data show that during pregnancy, as many as 70% of women demonstrate increased growth of inflammation-associated bacteria [18,27]. The growth of Firmicutes bacteria, including *Faecalibacterium* and *Eubacterium*, characterized by the production of C4:0, is observed. Proteobacteria and Actinobacteria overgrowth is also found to correlate with inflammatory bowel diseases and the level of pro-inflammatory cytokines [15,28,29]. It is suggested that features of the microbiota in early gestation may predict the pregnancy outcome [23]. Importantly, it has been shown that women who were overweight or obese before pregnancy (BMI ≥ 25) or in pregnancies complicated by gestational diabetes mellitus (GDM) had a reduced diversity of microorganisms [25]. There are data confirming that in nonpregnant hosts, the gut microbiota may also induce symptoms of metabolic syndrome [25]. The gut microbiota interacts with the host, altering the host physiological and metabolic processes. The gut microbiota plays a critical role in the development of obesity, insulin resistance, and type-2 diabetes as well. In patients with chronically impaired blood glucose response, the risk for type-2 diabetes appearance rises to 70% [22].

Thus, the number of microorganisms with anti-inflammatory properties, which are important in the production of butyrate, e.g., *Faecalibacterium*, decreases during pregnancy, as well as in metabolic disorders. These changes are considered to play a key role in the development of insulin resistance (IR) and weight gain in pregnant women in the last trimester. In pregnancy, SCFAs may also regulate inflammatory processes involved in labor and modulate inflammatory pathways in fetal membranes [6].

The possibilities of changing the body’s functions, both at the cellular and molecular level, are possible through G-coupled protein-coupled receptors 41 and 43 (GPR41 and GPR43), currently renamed for their function into free fatty acid receptors 2 and 3 (FFAR2 and FFAR3), and the histone deacetylase (HDAC) inhibitory mechanism. The FFAR2 receptor is activated mainly by acetate and propionate, while FFAR3 by the butyrate and propionate action [8,28]. The SCFA transporters in the organism are monocarboxylate transporter 1 (MCT-1) and sodium-coupled monocarboxylate transporter 1 (SMCT-1) [30]. The most important substrates for SCFA production are polysaccharides.

Summarizing the above, the content of SCFA depends on many factors, including diet, age, physiological state, genotype of the organism and, above all, the state of the intestinal microflora [14,29]. This work focuses on changes in the prevalence of microbiota metabolites, i.e., SCFAs, during pregnancy with an emphasis on its initial stage. The authors decided to determine whether and how SCFAs change during pregnancy as well as to analyze their importance for the metabolic parameters of carbohydrate metabolism, including body weight, BMI, and weight gain during pregnancy. This little-known but significant aspect will deepen the knowledge and create new possibilities for pregnant women and their offspring attending primary care clinics.

## 2. Results

The average daily dietary nutrient intake within two days before stool sampling did not differ significantly between the analyzed groups (OW and CG), except for glucose levels. Trends of lower fructose and fiber consumption in the obese group were visible (Table 1).

The SCFA profile in the OW group differed significantly from that in the CG, as shown in Table 2. Statistically significant higher levels were observed in relation to: C3: 0 (propionic acid), in the OW group and a reduced rate of C6: 0 (caproic acid) in the OW group in relation to the CG. There was also a decreased tendency of linear C5: 0 (valeric acid) in the OW group (Table 2).

Analyzing the correlations of anthropometric parameters in all pregnant women, no significant relationship was demonstrated (Table 3). A significant positive correlation between C3:0 and the glucose level was observed at 0 min for OGTT and glycated hemoglobin (HbA1c). Moreover, correlations with biochemical measurements also showed negative correlations of C 4: 0 n and C 6: 0 n (linear) with the glucose level at 0 min with respect to the OGTT and insulin level as well (Table 3).

Because we assumed that the individual dependencies could mask each other, the next step was to determine the relationship between the anthropometric and biochemical parameters and the SCFA concentrations in the OW and CG groups (Table 4 and Table 5). In the group of women who were overweight or obese before pregnancy, we found a positive correlation between C2:0 with nearly all anthropometric and biochemical parameters except the week of pregnancy, body weight gain, and glucose levels in the OGTT at 120 min (Table 4). C3: 0 was found to correlate positively with body weight gain during pregnancy, HbA1 levels and glucose levels in the OGTT at 0 min. The remaining anthropometric parameters did depend on C 4:0 n (linear) in the OW group (Table 4). Additionally, the C5:0 n (linear) levels were related to the week of pregnancy, altered glucose levels at 0 min in the OGTT, and glycated hemoglobin (Table 4).

A similar relationship with the parameters of carbohydrate metabolism was found in the group of women with normal body weight (Table 5). C2:0, C3:0, and C4:0n (linear) showed a correlation with anthropometric measurements. Moreover, C2:0, C3:0, branched, and C4:0n (linear) were correlated with the parameters of carbohydrate metabolism. Negative correlations of C4:0n and C5:0n (linear) with insulin levels and body weight gain, respectively, were observed (Table 5).

## 3. Discussion

A correlation between the SCFA produced by the anaerobic fermentation of carbohydrates in the colon and the parameters related to the metabolism of pregnant women and their anthropometric parameters was observed. At the same time, no major differences in nutrient and dietary fiber intake were found. Therefore, it should be concluded that the levels of SCFAs were mainly associated with the gut microbiota in the studied patients although there was a noticeable trend of lower fiber and higher glucose consumption in the group of obese women. Both nutrients are essential for the development of dysbiosis and the production of SCFAs [31]. In the research of Rahat-Rozenbloom et al., the investigators analyzed the effects of SCFAs on carbohydrate metabolism. It has been proven that the increase in colonic propionate stimulates both the insulin secretion in β-cells and glucagon-like peptide 1 (GLP-1) activity. In addition, propionate, with long-term action, protects β-cells from apoptosis induced by pro-inflammatory cytokines and free fatty acid (FFA) activity [32]. Moreover, a longer period of propionate production was also associated with faster elevation of the plasma insulin concentration, 30 min after a meal. SCFAs transmit signals in such a way that they bind to the free fatty acid receptors 2 and 3 (FFAR2 and FFAR3). Propionate has been found to stimulate insulin secretion in pancreatic cells via FFAR2. Higher FFAR2 activity was detected in the endocrine pancreas compared to its exocrine portion. According to the research studies, the stimulating effect of propionate on insulin secretion in human pancreatic islets depends on the protein kinase C (PKC) activation. C3:0 acid has also the ability to participate in the tricarboxylic acid (TCA) cycle. Thus, it can secrete larger amounts of insulin by increasing the synthesis of adenosine triphosphate (ATP) [18]. In our study, no such effect could be observed.

It has been demonstrated in animal models that a high-cholesterol diet is associated with significant changes in SCFA levels by affecting butyrate and propionate production and an increase in LPS secretion [32,33]. However, in our own studies, cholesterol consumption did not differ significantly between the groups. In our research, an increase in propionate concentration was observed in the group of obese women compared to the control group, which could be a confirmation of one possible cause of increased insulin levels occurring in the later weeks of pregnancy. However, due to the statistically insignificant differences between the groups, no relationship between C3:0 acid and insulin concentrations was found, although an association with blood glucose level at 0, 120 min for OGTT and glycated hemoglobin was observed.

It seems that obese women before pregnancy are more likely to suffer from pathological disturbances associated with intestinal permeability, possibly caused by intestinal dysbiosis [33,34]. Moreover, C5:0 acid, which decreased in the group of obese women before pregnancy, is also a good marker of liver fat accumulation and may predict the occurrence of type 2 diabetes [35].

Generally, in the group of pregnant women, the most significant relationships were found for C3:0, C4:0 n, and C6:0n acids, where the linear acids had a negative correlation. A positive correlation between C3:0, glycated hemoglobin, and fasting glucose levels confirmed the findings of other researchers, suggesting that propionate could set the precedent for the treatment of obesity [17,26]. Propionate may play a significant role in obesity, where non-esterified fatty acids (NEFAs) and pro-inflammatory cytokines trigger pancreatic β-cell dysfunction and apoptosis [29]. The increase in propionic acid levels with obesity and the correlation with glycosylated hemoglobin and fasting glucose levels in our research should be considered a probable factor of glucose metabolism disorders in pregnant women.

Following these lines of deductive reasoning, the C6:0 (linear) acids appear to be negatively related to the insulin level. Due to the lower rate of C6:0 (linear) acid in the total SCFA pool, it might be worthwhile to use this organic compound for management of excessive weight gain, but no relevant literature has been found on this subject.

However, it has been proven that GDM diagnosed in the later stages of pregnancy is associated with an inappropriate composition of the microbiota at the time of diagnosis [5]. The growth of *Bifidobacteria* correlates with increased glucose tolerance, regulates IO and insulin secretion, and helps reduce inflammation. SCFAs influence glycemia through glucagon-like peptide 1 (GLP-1) and pancreatic polypeptide (PPY), which are intestinal hormones. The intestinal hormone PYY is a peptide that acts as a paracrine substance to stimulate the feelings of satiety or hunger in the control center. In addition, it stimulates the action of insulin in muscles and adipose tissue. In a study in which colonocytes were collected from the colon of humans and rats, FFAR2 and FFAR3 receptors were linked to PYY-secreting L cells. In an animal model, after intracolonic SCFA infusions, an increase in the body’s PYY level was observed [10]. In diabetes, an increased level of tissue plasminogen activator inhibitor-1 (PAI-1) is also observed, which correlates with hypertension and microbiota composition responsible for butyrate production as well. Women diagnosed with GDM had higher arterial blood pressure values [3].

The group of bacteria to which microorganisms of the genera *Streptococcus*, *Bifidobacterium*, *Escherichia* and *Lactobacillus* belong affects the synthesis of neurotransmitters in the nervous system. Their imbalance may contribute to increased vessel tension, their narrowing, increased peripheral resistance, and subsequently to arterial hypertension development [36]. Moreover, the role of butyrate and the enzyme butyrate kinase by intensifying the action of PAI-1 in the prevention of the development of arterial hypertension during pregnancy is emphasized by other investigators [36]. The number of bacteria responsible for butyrate production and the number of copies of the butyrate kinase enzyme are inversely correlated with systolic and diastolic blood pressure and PAI-1, the marker of inflammation in women who are overweight and obese during pregnancy [3]. It is reported that taking probiotics is helpful in lowering blood pressure [37]. Taking into consideration the fact that acetate constitutes the main SCFA pool (about 37%), correlations of this acid concentration between the pre-pregnancy obese and normal-weight women groups were checked. We demonstrated an influence of acetate on anthropometric parameters in both groups of women. However, the composition of the gut microbiota has not been studied, which is a limitation of the study.

Thus, acids may be the first component elements of the conceptual puzzle changing the pathways of carbohydrate metabolism, focused on the mechanisms associated with the accumulation of reserves in order to survive, the disturbance of which destroys one of the first homeostasis barriers in the body.

Research studies on preeclampsia (PE), which is related to the immune system, suggest a significant reduction in the level of acetate in the blood serum of pregnant women [38]. We observed such a correlation, especially in the group of obese women who were more likely to develop PE before pregnancy. In a study on germ-free mice, as much as a 30% increase in acetate contributed to reducing the risk of PE. In addition, acetate is an important factor influencing blood pressure regulation, a disorder of which leads to PE clinical features [39].

An interesting observation coming from our research is the fact that linear SCFAs participate in the regulation of carbohydrate metabolism regardless of obesity. Dietary fiber-related SCFAs are involved in the regulation of carbohydrate metabolism, while BSCFAs, isobutyric, isovaleric, and isocaproic acid, from fermentation of undigested protein reaching the colon, do not have such an effect.

## 4. Materials and Methods

### 4.1. Study Group

The study was approved by the Ethical Committee of Pomeranian Medical University (approval code: KB-0012-69-18; approval date: 18/06/2018), and informed consent was obtained from all individual participants included in the study. The study group consisted of 90 Caucasian women, mean age 32.17 ± 5.32 years. The diagnosis of pregnancy was confirmed based on sensitive biochemical assays and high-resolution ultrasonography (General Electric, Voluson E8 Expert, 2015) and women from the 10th week of gestation were included in the study. The exclusion criteria were twin pregnancies, refusal of consent for examination, the presence of active infection or neoplastic disease. The characteristics of the study group are presented below in Table 6.

In order to eliminate the influence of the obesity-associated chronic states and to analyze the impact of pregnancy on the SCFA levels, the study group was divided into obese patients and patients with normal BMI before pregnancy. The first group consisted of 48 women who had an overweight and obese pre-pregnancy BMI over 30 (OW); the second group, the control group (CG), consisted of 42 women with normal body weight, as shown in Table 7.

A 24-h consumption interview was conducted twice. The interviews were done two days before collecting a stool sample. To provide information regarding the size of the consumed meals, an album of products and meal photos was used. The menus were analyzed using quantitative methods. The Dieta 6D program, recommended by the National Centre of Nutritional Education (Warsaw, Poland), was used. The acquired data were compared between groups.

### 4.2. Sampling

#### 4.2.1. The Blood Chemistry Parameters

The blood samples (approximately 5 mL) were taken from a vein in the arm in the morning strictly on an empty stomach after a 10–12-h break in meals. Biochemical parameters of carbohydrate metabolism (glucose, insulin, glycosylated hemoglobin) were performed using standard laboratory methods in an accredited hospital laboratory using the Roche Diagnostic Cobas e411 and Cobas Integra B Plus analyses. An oral glucose tolerance test (OGTT) was performed on women who had a fasting glucose level in blood below 100 mg/dL and consented; 75 g of glucose was administered to drink and its level was measured after 60 and 120 min (Table 2). No women took metformin or insulin. The obese group (OW) consisted of 20 patients (normal glucose tolerant (*n* = 11), impaired glucose tolerance (*n* = 3), impaired fasting glucose (*n* = 1), and impaired fasting glucose + impaired glucose tolerance (*n* = 5)), and the control group (CG) consisted of 15 normal glucose-tolerant patients.

#### 4.2.2. Collecting a Stool Sample

All study participants were asked to collect a stool sample into a screw-capped collection container and demanded not to use laxatives and change the diet. Women sampled feces after overnight fasting, to establish a common reference point for food intake, and delivered these to the office within 24 h (a factor determining SCFA synthesis). After the stool collection, the samples were immediately frozen at −20 °C and transported on ice to our laboratory, then stored at −80 °C until the analyses. We carried out the analysis within six months after the fecal sample collection [40].

### 4.3. Isolation of SCFAs

A 0.5 g fecal sample was suspended in a tube containing 5 mL of distilled water and mixed intensively for 5 min by used shaker. Using 5 M HCl solution, the pH of suspension was adjusted to 2–3. The samples were then shaken for 10 min and centrifuged for 20 min at 5000 rpm. Subsequently, the supernatant was filtered (Ø 400 µm filter) and transferred to a chromatographic vial for gas chromatography analyses [40].

### 4.4. Gas Chromatography SCFAs

The following SCFAs were analyzed: acetic acid (C 2:0), propionic acid (C 3:0), isobutyric acid (C 4:0 i), butyric acid (C 4:0 n), isovaleric acid (C 5:0 i) valeric acid (C 5:0 n), isocaproic acid (C 6:0 i), caproic acid (C 6:0 n). Chromatographic analyses were carried out using the Agilent Technologies 1260 A GC system with a flame ionization detector (FID). A fused-silica capillary column with a free fatty acid phase (DB-FFAP, 30 m × 0.53 mm × 0.5 um) was used. The carrier gas was hydrogen at a flow rate equal to 14.4 mL/min. The initial temperature (100 °C) was maintained for 0.5 min, then raised to 180 °C with ramping of 8 °C/min to be constant for 1 min. Subsequently, the temperature was increased to 200 °C (ramping 20 °C/min), to finally reach 200 °C and then sustained for 5 min. The injection volume was 1 μL and the run time of a single analysis was 17.5 min. Fatty acids were identified by comparing their retention times with those of commercially available standards [41].

### 4.5. Statistical Analyses

Statistical analyses were performed with Statistica 13.3 software (Statsoft, Cracow, Poland). The Shapiro–Wilk parametric test was used (the distribution in most cases was normal). A non-parametric test was used: Mann–Whitney test for comparisons between groups (obese women before pregnancy and control group); *p* < 0.05 was considered statistically significant. Then, we correlated the SCFA percentage and the biochemical parameters of carbohydrate metabolism and anthropometric measurements in women.

## 5. Conclusions

In conclusion, we report that SCFAs may at least partially contribute in the course of pregnancy and participate in the alteration of carbohydrate metabolism. The influence of acetic acid during pregnancy on some anthropometric parameters was visible in both groups. Linear butyrate, valeric and especially propionate regulate glucose metabolism by stimulating the process of intestinal gluconeogenesis. The level of propionic acid decreases with the course of pregnancy, while its increase is characteristic of obese women, which is associated with many metabolic adaptations. Linear caproic acid and propionic acid levels can be an important critical point in maintaining lower anthropometric parameters during pregnancy.

## Figures and Tables

**Table 1:** Average consumption of nutrients in the diet in both groups.

	OW Avg ± SD	CG Avg ± SD	*p*-Value
Protein (%)	16.52 ± 3.1	14.72 ± 2.81	0.157
Vegetable (g)	29.37 ± 5.79	35.13 ± 6.72	0.301
Animal (g)	64.95 ± 17.82	68.49 ± 15.61	0.416
Fat (%)	38.31 ± 7.09	39.27 ± 7.96	0.431
Cholesterol (mg)	221.52 ± 104.23	245.64 ± 132.4	0.184
Carbohydrates (%)	43.23 ± 8.29	43.96 ± 9.02	0.649
Dietary fibre (g)	18.33 ± 5.21	21.41 ± 7.37	0.091 *
Glucose	64.86 ± 15.62	34.89 ± 12.46	**0.044**
Lactose	13.85 ± 4.62	12.31 ± 5.32	0.693
Fructose	12.71 ± 5.29	16.98 ± 6.31	0.083 *
Saccharose	62.65 ± 12.47	56.72 ± 10.12	0.316
Starch	138.42 ± 27.18	162.76 ± 32.84	0.485

The bold text—significant; *—trend towards significance.

**Table 2:** Comparison of the SCFAs in the stool in the analyzed study groups of women (OW and CG).

SCFAs (%)	Total (*n* = 90)	OW Avg ± SD	CG Avg ± SD	*p*-value
C 2:0 acetic	36.94 ± 7.69	36.93 ± 9.22	36.95 ± 8.54	0.991
C 3:0 propionic	19.63 ± 6.08	20.17 ± 6.80	17.33 ± 3.91	**0.022**
C 4:0 i butyric (branched)	5.38 ± 3.44	5.55 ± 4.48	5.22 ± 1.80	0.569
C 4:0 n butyric (linear)	21.37 ± 8.18	21.28 ± 8.23	21.48 ± 8.52	0.915
C 5:0 i valeric (branched)	8.32 ± 4.02	7.87 ± 4.06	8.86 ± 3.93	0.255
C 5:0 n valeric (linear)	5.11 ± 2.27	4.62 ± 1.86	5.58 ± 2.67	0.055 *
C 6:0 i caproic (branched)	0.36 ± 0.47	0.41 ± 0.61	0.32 ± 0.25	0.384
C 6:0 n caproic (linear)	1.90 ± 1.74	1.46 ± 1.55	2.30 ± 1.8	**0.023**

The bold text—significant; *—trend towards significance.

**Table 3:** Analysis of the relationship between SCFAs in the stool and the overall parameters in the groups of pregnant women.

Parameters	C 2:0	C 3:0	C 4:0 i (Branched)	C 4:0 n (Linear)	C 5:0 i (Branched)	C 5:0 n (Linear)	C 6:0 i (Branched)	C 6:0 n (Linear)
Week of pregnancy	0.034	−0.073	0.004	0.043	0.027	−0.035	0.235	−0.118
Body weight before pregnancy (kg)	−0.026	0.115	0.045	0.073	0.010	−0.124	0.180	−0.333
BMI before pregnancy	0.080	0.007	−0.024	0.106	−0.044	−0.192	0.177	−0.369
Body weight during pregnancy (kg)	−0.022	0.102	0.025	0.094	0.004	−0.133	0.204	−0.353
BMI during pregnancy	0.095	−0.013	−0.047	0.127	−0.052	−0.207	0.208	−0.398
Body weight gain (kg)	0.032	−0.103	−0.118	0.084	−0.038	−0.002	0.063	0.017
Insulin (mU/mL)	0.342	0.000	−0.147	−0.088	−0.085	−0.246	0.261	**−0.455**
HbA1c (%)	−0.247	**0.443**	0.185	−0.236	0.266	0.228	−0.085	−0.147
Fasting glucose (mg/dL)	−0.072	**0.473**	0.172	**−0.420**	0.271	0.205	0.296	−0.260
OGTT at 60 min (mg/dL)	0.013	−0.187	−0.367	0.290	−0.252	−0.066	−0.122	0.238
OGTT at 120 min (mg/dL)	0.023	0.193	−0.021	−0.310	0.099	0.140	0.243	0.155

Bold text—significant.

**Table 4:** Analysis of the relationship between SCFA in the stool and the parameters in the group of overweight and obese pregnant women (OW).

Parameters	C 2:0	C 3:0	C 4:0 i (Branched)	C 4:0 n (Linear)	C 5:0 i (Branched)	C 5:0 n (Linear)	C 6:0 i (Branched)	C 6:0 n (Linear)
Week of pregnancy	0.233	0.387	0.421	0.215	0.515	**0.597**	0.011	−0.095
Body weight before pregnancy (kg)	**0.651**	0.539	0.009	**0.641**	0.137	0.214	−0.342	−0.176
BMI before pregnancy	**0.728**	0.405	−0.114	**0.690**	0.036	0.119	−0.340	−0.213
Body weight during pregnancy (kg)	**0.649**	**0.565**	0.035	**0.658**	0.179	0.277	−0.363	−0.142
BMI during pregnancy	**0.729**	0.430	−0.088	**0.707**	0.080	0.185	−0.360	−0.180
Body weight gain (kg)	−0.364	−0.057	0.187	−0.105	0.251	0.366	−0.046	0.334
Insulin (mU/mL)	**0.654**	0.011	−0.508	0.378	−0.353	−0.217	−0.233	−0.185
HbA1c (%)	**0.686**	**0.678**	0.147	0.484	0.355	**0.562**	−0.353	0.055
Fasting glucose (mg/dL)	**0.615**	**0.774**	0.226	0.339	0.434	**0.559**	−0.242	−0.118
OGTT at 60 min (mg/dL)	**0.573**	0.527	−0.084	0.510	0.150	0.406	−0.413	−0.042
OGTT at 120 min(mg/dL)	0.347	**0.592**	0.210	−0.143	0.401	0.423	0.129	−0.102

Bold text—significant.

**Table 5:** Analysis of the relationship between SCFAs in the stool, anthropometric parameters, and the carbohydrate metabolism parameters in the control group (CG).

Parameter	C 2:0	C 3:0	C 4:0 i (Branched)	C 4:0 n (Linear)	C 5:0 i (Branched)	C 5:0 n (Linear)	C 6:0 i (Branched)	C 6:0 n (Linear)
Week of gestation	0.609	0.296	0.038	0.467	−0.099	−0.201	0.480	−0.128
Body weight before pregnancy (kg)	**0.913**	**0.846**	0.608	**0.704**	0.412	0.611	0.159	0.045
BMI before pregnancy	**0.865**	**0.870**	0.631	0.628	0.463	0.612	0.176	−0.018
Body weight during pregnancy (kg)	**0.949**	**0.796**	0.525	**0.721**	0.327	0.464	0.289	−0.015
BMI during pregnancy	**0.927**	**0.836**	0.565	**0.667**	0.390	0.483	0.320	−0.076
Body weight gain (kg)	−0.085	−0.436	−0.480	−0.194	−0.422	**−0.716**	0.518	−0.275
Insulin (mU/mL)	−0.313	−0.230	−0.079	**−0.669**	0.140	−0.282	0.629	−0.503
HbA1c (%)	**0.898**	**0.888**	0.628	**0.748**	0.425	0.582	0.139	0.009
Fasting glucose (mg/dL)	**0.927**	**0.811**	0.519	**0.661**	0.343	0.543	0.271	0.055
OGTT at 60 min (mg/dL)	**0.730**	0.470	0.252	**0.811**	0.094	0.371	0.240	0.331
OGTT at 120 min(mg/dL)	**0.781**	0.536	0.270	**0.816**	0.090	0.417	0.181	0.362

Bold text—significant.

**Table 6:** Characteristics of the study group of women (*n* = 90).

Parameter	Avg	SD
Age (years)	32.17	5.32
Height (m)	1.68	0.05
Body weight before pregnancy (kg)	85.19	20.69
Body weight during pregnancy (kg)	89.64	19.73
BMI before pregnancy (kg/m^2^)	29.42	8.45
BMI during pregnancy (kg/m^2^)	31.36	7.53
Week of gestation (week)	20.64	7.73
Fasting insulin (mU/mL)	15.79	14.02
HbA1 (%)	5.11	0.32
Fasting glucose (mg/dL)	89.46	11.82
OGTT at 60 min (mg/dL)	134.78	28.33
OGTT at 120 min (mg/dL)	114.52	29.59

**Table 7:** Division of the study group of women.

Parameter	OW Avg ± SD	CG Avg ± SD	*p*-Value
Age (years)	30.26 ± 5.21	32.34 ± 5.14	NS
Height (m)	1.68 ± 0.06	1.67 ± 0.05	NS
Body weight before pregnancy (kg)	100.11 ± 19.17	66.93 ± 13.98	0.0000004 *
Body weight during pregnancy (kg)	102.42 ± 20.01	72.29 ± 15.05	0.0001 *
BMI before pregnancy (kg/m^2^)	34.44 ± 8.52	22.72 ± 6.62	0.00001 *
BMI during pregnancy (kg/m^2^)	35.21 ± 8.78	25.17 ± 6.18	0.012 *
Weight gain (kg)	2.33 ± 4.21	7.23 ± 9.67	0.004
Week of gestation (week)	19.93 ± 6.49	21.82 ± 7.13	NS
Fasting insulin (mU/mL)	16.31 ± 8.08	12.77 ± 10.82	NS
HbA1 (%)	5.06 ± 0.84	4.88 ± 0.83	NS
Fasting glucose (mg/dL)	92.36 ± 20.57	82.91 ± 11.01	NS
OGTT at 60 min (mg/dL)	136.01 ± 29.22	126.51 ± 27.03	NS
OGTT at 120 min (mg/dL)	117.59 ± 34.17	114.43 ± 23.99	NS

NS—not significant; * *p*-value (1 × 10^−5^).
